# Seizures and Consciousness Disorder Secondary to Intracranial Hypotension After Spinal Surgery: A Case Report and Literature Review

**DOI:** 10.3389/fneur.2022.923529

**Published:** 2022-06-27

**Authors:** Yuqing Lv, Hui Xiang

**Affiliations:** ^1^Department of Gastroenterology, Zhongnan Hospital of Wuhan University, Wuhan, China; ^2^Department of Critical Care Medicine, Zhongnan Hospital of Wuhan University, Wuhan, China

**Keywords:** seizure, intracranial hypotension, status epilepticus, cerebrospinal fluid leakage, spinal surgery, critical care

## Abstract

**Rationale:**

Cerebrospinal fluid (CSF) leakage is a common condition after spinal surgery and is also the most common cause of intracranial hypotension. Intracranial hypotension (IH) is typically characterized by an orthostatic headache with associated nausea, vomiting, tinnitus, vertigo, hypoacusis, neck stiffness, and photophobia. There have been limited case reports describing surgery-associated IH presenting with seizures and disorder of consciousness. Due to the atypia of symptoms, these clinical manifestations are usually ignored or even misdiagnosed. As a result, clinicians face a significant challenge in detecting IH early and understanding its various clinical presentations. Meanwhile, we summarize the cases of IH presenting as seizures in recent years, including its clinical characteristics and effective treatment, which will be very helpful for the early diagnosis of IH.

**Patient concerns:**

A 72-year-old Chinese male patient developed status epilepticus, a disorder of consciousness, and quadriplegia when he finished spinal surgery, although he had no previous seizures or any seizure risk factors.

**Diagnosis:**

After MRI and CT examination, subdural hygromas were found under both sides of the skull, and combined with the clinical manifestations of the patient, intracranial hypotension due to cerebrospinal fluid leakage was diagnosed.

**Interventions:**

In the early stage, we carried out strict perioperative critical care for the patient. Trendelenburg position was conducted to relieve intracranial hypotension. The dural repair surgery was performed after the diagnosis of CSF leakage.

**Outcomes:**

Seizures in the patient were resolved. Three months after discharge, he was gradually returning to normal life.

**Lessons:**

One possible cause of unexplained seizures and disorder of consciousness after spinal surgery is cerebrospinal fluid leakage associated with intracranial hypotension syndrome. Trendelenburg position and dural repair surgery are effective ways to relieve intracranial hypotension and associated symptoms. Better awareness of the association between IH (intracranial hypotension) and seizures may help us improve early recognition of the syndrome.

## Introduction

The syndrome of intracranial hypotension has been increasingly diagnosed since the introduction of magnetic resonance imaging (MRI), and its incidence is now about 5 per 1,00,000 of the population (1). Cerebrospinal fluid (CSF) leakage is the most common cause of intracranial hypotension. Defects along the dura (including tears and fistulas), leading to CSF leakage, can be congenital and traumatic in etiology (2), with surgery being the most common traumatic cause. Intracranial hypotension (IH) typically presents with a postural headache with associated nausea, vomiting, tinnitus, vertigo, hypoacusis, neck stiffness, and photophobia (2–5), while seizures and the disorder of consciousness are rare. Therefore, the disorder of consciousness and seizures after spinal surgery are of concern because they may be associated with intracranial hypotension caused by cerebrospinal fluid leakage.

## Ethics Statements

Informed written consent was obtained from the patient's son after the nature of the study had been fully explained to them. The patient's son provided informed consent for the publication of the case.

## Case Presentation

A 72-year-old Chinese male patient developed seizures, disorder of consciousness, and quadriplegia when he finished spinal surgery. Due to 3 years of pain and numbness in both lower limbs, he underwent spinal surgery, including L3/4/5 posterior decompression, interbody fusion, and internal fixation, plus L2/3 left lamina decompression. His medical history was significant hypertension for about 8 years and he usually took amlodipine, an oral antihypertensive drug. Therefore, his blood pressure was well-controlled. He had no previous seizures or any seizure risk factors.

The operation went smoothly and the patient soon regained consciousness. Then, the endotracheal tube was safely removed. In the surgical resuscitation room, the patient complained of itchy skin and generalized clonic seizures, which became progressively worse, characterized by bilateral clonic movements in the upper and lower limbs. Profound tachycardia and hypertension accompanied the spell. Therefore, the patient was transferred to the intensive care unit (ICU) immediately and tracheal intubation was performed instantly. Subsequently, consciousness disturbance occurred with blood oxygen saturation declining, and the ECG (electrocardiograph) monitor displayed that there was an atrial fibrillation rhythm and it quickly developed into ventricular fibrillation. He was given timely shock defibrillation, after which sinus rhythm was regained, with Norepinephrine maintaining blood pressure. Meanwhile, the emergency blood gas analysis suggested a pH of 6.9 and a pCO_2_ of 95 mmHg. For emergency seizure control, intravenous midazolam was given. In addition, an extremely high level of creatine kinase and myohemoglobin was found in his blood test, which was thought to be the result of a violent convulsion. We gave the patient rocuronium bromide, a muscle relaxant, to prevent further muscle damage.

**Figure 1:**
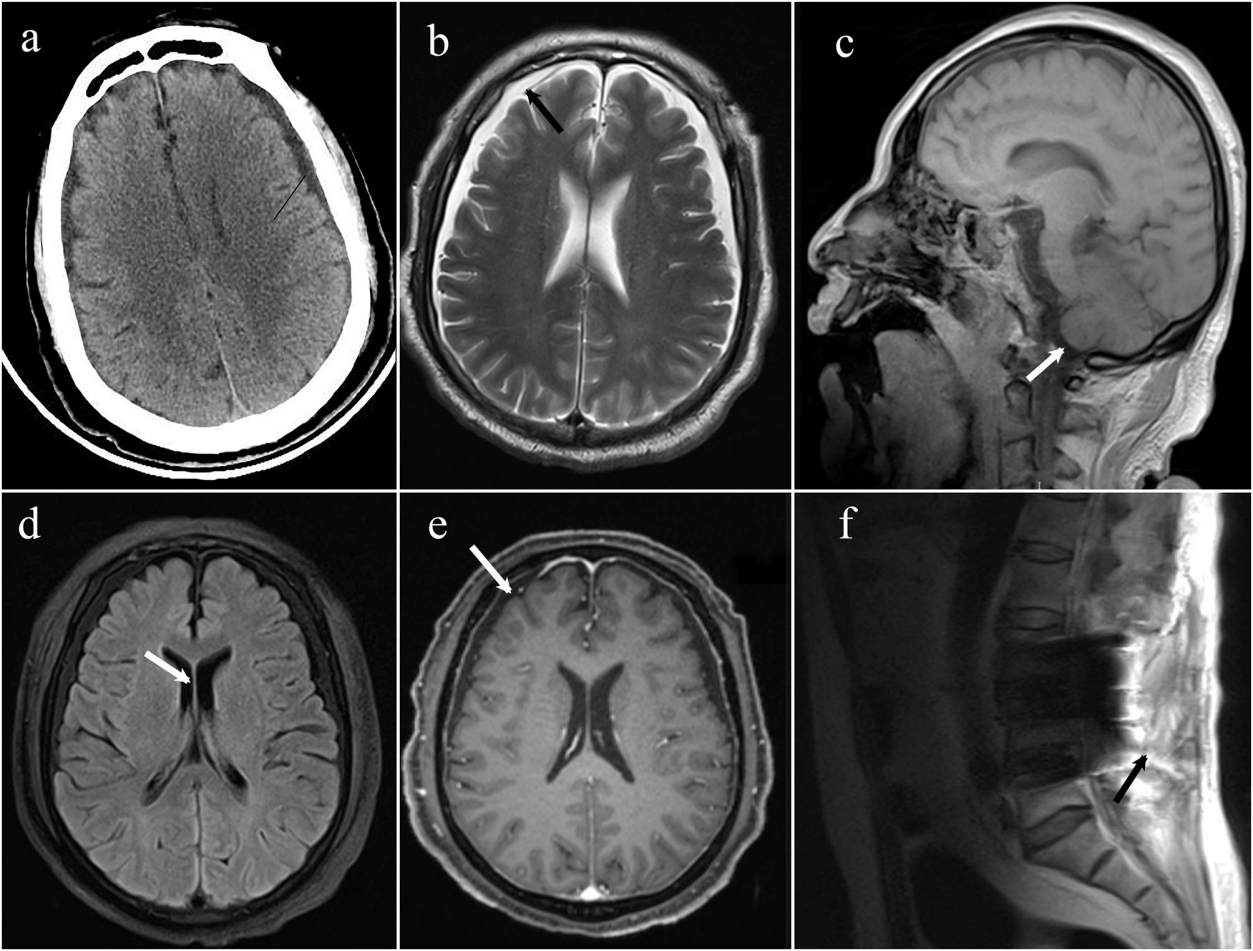
**(a)** The emergency CT suggested that the space under the inner plate of the skull was widened on both sides, which was considered a subdural effusion; **(b)** axial T2 weighted MRI demonstrates bilateral subdural effusions (arrows); **(c)** sagittal T1-weighted MRI demonstrates sagging of the brain; **(d)** axial T1 weighted MRI demonstrates bilateral ventricles narrowing; **(e)** axial T1 post-gadolinium showing diffuse, mild pachymeningeal thickening, and enhancement (arrow); **(f)** MRI spine sagittal T2 sequence demonstrates a fluid collection within the posterior paraspinal soft tissue.

An emergency brain CT (computerized tomography) was conducted, suggesting that the space under the inner plate of the skull was widened on both sides, which was considered a subdural effusion (Figure 1). No obvious signs of cerebral hemorrhage and cerebral infarction were found. After first aid, we conducted target temperature management for the patient and implemented a hypothermia strategy to maintain its core temperature between 32 and 34°C. Levetiracetam and midazolam were added to abort seizures as the disorder of consciousness and seizures persisted. Norepinephrine and dopamine were used to achieve a mean arterial pressure >65 mmHg, with a maximum of 1 μg/kg/min for norepinephrine and 15 μg/kg/min for dopamine. Blood oxygen saturation fluctuated around 85% at pure oxygen concentration due to the presence of refractory hypoxemia. On the second day of ICU admission, he was given a tracheostomy and ventilator-assisted ventilation to maintain oxygen saturation >94% and pCO_2_ between 35 and 45 mmHg. Given his lack of gastrointestinal hemorrhage and intestinal dysfunction, enteral nutrition was administered within 48 h, along with insulin to keep his blood sugar at 8–10 mmol/L.

Three days after the symptom onset, the use of vasoactive agents and sedatives such as midazolam were suspended, and a significant improvement in convulsions was observed, as well as blood oxygen saturation was maintained above 94% at 35 oxygen concentration, which was an indication that respiration and epilepsy were getting better. Simultaneously, blood pressure also returned to normal, at around 140/60 mmHg, thus the use of epinephrine was interrupted. As the patient's condition stabilized, we performed an EEG for 24 h, which revealed a diffuse slow waves background with a large number of fast waves, but we did not pick up the epileptic waves. Whereas, we observed that more than 350 ml of hemorrhagic fluid could be drained from the drainage tube of the patient's waist wound every day, and he was still in a comatose state without an obvious response to pain stimuli. We reviewed the blood concentration of valproate and it was in the normal range. A CT scan of the brain was also reviewed, indicating there was mild cerebral edema and ventricular shrinkage. The existence of cerebrospinal fluid leakage was highly suspected and it was considered that the patient's consciousness disturbance and epileptic seizure might be related to it because it can lead to intracranial hypotension. We adopted a Trendelenburg position to prevent the excessive outflow of cerebrospinal fluid. Sure enough, after changing the patient's position, his disturbance of consciousness improved. Simultaneously, he was able to respond to verbal stimuli and partially follow movements. Over the next few days, his cerebrospinal fluid drainage gradually decreased to about 200 ml per day.

**Figure 2:**
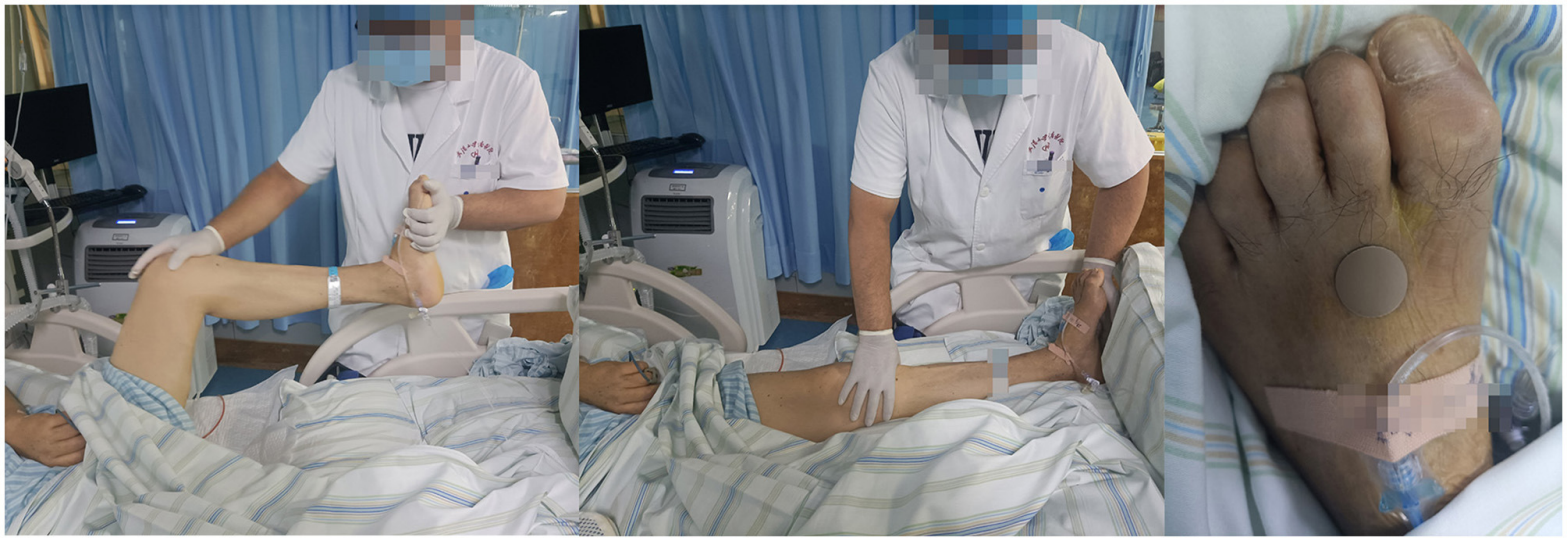
The first two pictures show that the neuro-rehabilitation doctor is giving him muscle massages. The third picture shows the special treatment of Traditional Chinese medicine, acupoint acupuncture.

On day 10, the patient was successfully removed from the ventilator, so he underwent MRI and enhanced MRI of his brain and spinal cord, revealing evidence of bilateral subdural effusion and mild inferior hernia of the bilateral cerebellar tonsil, bilateral ventricles narrowing, pachymeningeal enhancement, which is consistent with intracranial hypotension. Several tiny hemorrhagic foci were also in the left cerebellar hemisphere and right temporal lobe. Spinal MRI demonstrated postoperative changes of the lumbar spine, lumbosacral fascia edema, and fluid collection between the intrathecal site and the soft tissue; however it did not identify the site of cerebrospinal fluid (CSF) leakage (Figure 2). These changes in the brain were indicative of typical intracranial hypotension, such as subdural effusion, mild inferior hernia of the bilateral cerebellar tonsil, ventricular narrowing, and pachymeningeal enhancement which could be caused by cerebrospinal fluid leakage.

Taking all the evidence together, we strongly suspected that he had a cerebrospinal fluid leak. Therefore, we contacted an orthopedic surgeon for dural repair and wound debridement and a drainage tube was placed in the operation area. During the operation, CSF leakage was proved to be present. Thus, intracranial hypotension due to cerebrospinal fluid leakage secondary to intraoperative dural tear was diagnosed.

After surgery, we still kept the Trendelenburg position. On the other hand, a neuro-rehabilitation doctor was brought in for muscle massage and acupuncture. Over the next few days, he gradually regained consciousness and was able to follow instructions better, accompanied by a gradual reduction of clear to bloody spinal drainage fluid. At the same time, his upper body strength was gradually recovering to level 2. The drainage tube was removed when the drainage fluid was <50 ml for more than 2 days. As the condition gradually improved, the patient's family requested that he be transferred back to the local hospital for further rehabilitation. We followed him up for 3 months. A month after discharge, with his independent diet and defecation, we learned that he had complete sensation in his limbs and level 3 strength in his lower extremities and that his trachea incision had been fully healed and closed. The only problem was the gastrointestinal disturbance, with bloating and constipation, which may be related to the neurotic disorder caused by the operation. Three months after discharge, his gastrointestinal function recovered well and he could walk about 50 m on a flat ground alone.

## Discussion

The syndrome of intracranial hypotension (IH) is caused by the leakage of CSF (cerebrospinal fluid) from the thecal sac within or along the spinal canal (3, 4). It is typically manifested by orthostatic headaches that may be associated with one or more of several other symptoms, including pain or stiffness of the neck, nausea, emesis, horizontal diplopia, dizziness, changes in hearing, visual blurring or visual field cuts, photophobia, interscapular pain, and occasionally face numbness or weakness or radicular upper-limb symptoms (5). Seizures caused by IH have also been observed in some rare clinical cases (6, 7). If the leakage can be arrested, it is potentially curable (3, 4, 8).

The most characteristic features of IH on intracranial MR (magnetic resonance) imaging include diffuse pachymeningeal enhancement and “brain sag” (9, 10). Sagging of the brain can cause a subdural hematoma or hygroma, kinking of the midbrain and pons toward the clivus, lessening of the distance (e.g.,) from the optic chiasm to the pituitary gland, and tension on the cranial nerves (3). Other features include dural contrast enhancement and enlargement of the pituitary gland. Homogenous contrast enhancement of the dura mater is the most sensitive sign (3).

Intracranial hypotension is usually treated with a progressive approach. Typically, headaches can be resolved with conservative management, which includes hydration and strict bed rest, allowing for relief of CSF pressure at the site of leakage and thus healing the underlying defect. Caffeine administered intravenously or orally is also effective for post-lumbar puncture IH. If a conservative approach is not effective, epidural blood patches, epidural saline infusion, and surgical correction should be considered (3, 4, 11). However, blood patches are more controversial when used for surgery-related dural leaks, although suggested to be safe in a case series because they can cause seizures and respiratory distress, or other related complications (12). Although the specific mechanism by which IH may cause seizure development and convulsive SE has not been fully elucidated, it has been proposed that IH may cause meningeal irritation or traction on cortical structures of the brain, and finally, result in seizures (6, 13). In addition, the alterations of intracranial pressure (ICP) may result in acute changes in cerebral blood flow, leading to seizure generation (14).

Intracranial hypotension presents in a variety of ways; however, reports of seizures secondary to IH are rare. We summarize the cases of IH presenting as seizures in recent years, including its clinical characteristics and effective treatment (Table 1). Gilmour et al. (7) describe a 71-year-old woman with chronic back pain who developed convulsive status epilepticus immediately after scoliosis surgery. MRI was consistent with IH, there was no cortical vein thrombosis, presumably due to an intraoperative dural tear causing status epilepticus, and the seizure terminated after bed rest. Lin et al. (13) describe an individual who had multiple seizures after a thoracic laminectomy and had imaging findings consistent with IH. A thoracic laminotomy was performed on this patient and a dural tear was found and repaired. Chaudhary et al. (6) describe cases of spontaneous intracranial hypotension causing focal onset seizures and impairment of awareness. Other two reports describe patients with hydrocephalus and ventriculoperitoneal shunts who had intractable epilepsy caused by overdrainage (14, 15). Delgado-López et al. (16) present a case of posterior reversible encephalopathy syndrome (PRES) following laminectomy and fixation for L4-5 lumbar stenosis and spondylolisthesis, characterized by status epilepticus, which is hypothesized by vasoconstriction, brain hypoperfusion, and cerebrospinal fluid. Pugliese et al. (17) report a case of a woman presenting headache and tonico-clonic seizures 7 days after epidural analgesia for a cesarean section. MRI showed alterations suggestive of the presence of intracranial hypotension (IH), as well as evidence of posterior reversible encephalopathy syndrome (PRES). They suggest that venous stagnation and hydrostatic edema, secondary to intracranial hypotension, probably played a crucial role in the pathogenesis of PRES. In our case, the patient developed seizures after spinal surgery, with imaging revealed bilateral subdural hygromas, which were thought to be related to intracranial hypotension syndrome. It is speculated that the existence of subdural hygromas may lead to the formation of hemorrhagic foci in brain, which felt to be acute, because of the pulling on the brain tissue. This may have caused the seizure due to the highly epileptogenic effect of blood. In addition, during the perioperative period, patients were given a large number of sedative drugs, such as midazolam, and whether these drugs are involved in the seizure is also a thought-provoking question.

**Table 1:** Summary of some reported cases of seizures secondary to intracranial hypotension (IH).

**Article**	**Patient presentation**	**Seizure description**	**The cause of IH**	**Treatment**
Gilmour et al. (7)	A 71-year-old woman with chronic back pain developed convulsive status epilepticus immediately after scoliosis surgery	It was characterized by bilateral clonic movements of her upper and lower extremities, with eyes open and a vertical upward tonic gaze deviation	An intraoperative dural tear secondary to elective redo-scoliosis surgery	Keep on strict bed rest
Lin et al. (13)	A 37-yr-old man with acute spinal cord compression at T9-10 because of pseudoarthritis developed generalized seizure after surgery	30 min postoperative generalized seizures lasting 15 s occurring every 10–15 min	Dural tear after laminectomy	Treated with midazolam, phenytoin, and dural tear repair
Chaudhary et al. (6)	A 60-year-old man who presented with a decreased level of consciousness developed a complex partial seizure involving left-sided facial twitching after the presentation	Focal motor-impaired awareness seizure with left-sided facial twitching	Small bilateral subdural hygromas	Treatment with a 20 cc blood patch in the lumbar spine
	A 37-year-old man who presented with a right-sided acute on chronic SDH which reaccumulated despite burr hole drainage, presenting with decreased level of consciousness	Focal impaired awareness and complex partial seizures	Chiropractic neck manipulation and trivial head trauma, potentially resulting in thoracic dural tear	A 20 cc autologous epidural blood patch was placed at the T12-L1 level
Delgado-López et al. (16)	An 82-year-old woman presented with a generalized tonic-clonic seizure after L4-5 laminectomy and decompression of the dural sac and origin of roots bilaterally	She developed transient hypotension for <1 min presented with a generalized tonic-clonic seizure that lasted 5 days	An unnoticed cerebrospinal fluid leakage secondary to surgery	Antiepileptic drugs, ventilators and other symptomatic support treatment
Pugliese et al. (17)	A 41-year-old woman presented a worsening of the headache and tonico-clonic seizures 7 days after epidural analgesia for a cesarean section	A worsening of the headache which had a gradual onset, was bilateral, pressure-like, with a postural component and tonico-clonic seizures with the left motor syndrome, mild right anisocoria, and rapid deterioration of the mental status	Inadvertent dural puncture during the epidural anesthesia	Treatment with support therapy followed by blood patch
Our report	A 72-year-old Chinese male patient developed seizures, disorder of consciousness, and quadriplegia when he finished spinal surgery	He developed itchy skin and generalized clonic seizures, characterized by bilateral clonic movements in the upper and lower limbs	Cerebrospinal fluid leakage secondary to the spinal surgery of L3/4/5 posterior decompression, interbody fusion, and internal fixation, plus L2/3 left lamina decompression	Treatment with support therapy, trendelenburg position, dural repair surgery, and traditional Chinese acupuncture and massage therapy

*IH, intracranial hypotension; SDH, subdural hematomas*.

Meanwhile, the patient did not develop typical postural headache symptoms but instead presented with a convulsive status epilepticus characterized by impaired limb movement and sensory function as well as impaired consciousness, which was also rare in previous reports. We adopted the Trendelenburg position treatment and a dural repair surgery, which proved to be very therapeutic. Gastrointestinal dysfunction occurred during the patient's subsequent recovery, and it is reasonable to speculate that this may be related to autonomic nervous dysfunction caused by the persistent epileptic state.

## Conclusion

Unexplained seizures and disorder of consciousness after spinal surgery may be associated with cerebrospinal fluid leakage associated with intracranial hypotension syndrome. Trendelenburg position and dural repair surgery are effective ways to relieve intracranial hypotension and associated symptoms. Better awareness of the association between IH and seizures may help us improve early recognition of the syndrome.

## Data Availability Statement

The original contributions presented in the study are included in the article/supplementary material, further inquiries can be directed to the corresponding author/s.

## Ethics Statement

Written informed consent was obtained from the individual(s) for the publication of any potentially identifiable images or data included in this article.

## Author Contributions

YL wrote the manuscript. HX revised the manuscript. All authors contributed to the article and approved the submitted version.

## Conflict of Interest

The authors declare that the research was conducted in the absence of any commercial or financial relationships that could be construed as a potential conflict of interest.

## Publisher's Note

All claims expressed in this article are solely those of the authors and do not necessarily represent those of their affiliated organizations, or those of the publisher, the editors and the reviewers. Any product that may be evaluated in this article, or claim that may be made by its manufacturer, is not guaranteed or endorsed by the publisher.

## Abbreviations

IH: intracranial hypotension
CSF: cerebrospinal fluid
SE: status epilepticus
ECG: electrocardiograph
PH: pondus hydrogenii
PCO_2_: partial pressure of carbon dioxide
MRI: magnetic resonance imaging
MR: magnetic resonance
ICU: intensive care unit
CT: computerized tomography
ICP: intracranial pressure
PRES: posterior reversible encephalopathy syndrome.
